# Identification and sequencing of temperature sensitive alleles of the Anaphase Promoting Complex component *mat-3* in *C. elegans*

**DOI:** 10.17912/micropub.biology.000889

**Published:** 2023-08-07

**Authors:** Katherine A. Maniates, Yamei Zuo, Kendall Flanagan, Shania G. Halim, Alexander Kane, Andrew Singson

**Affiliations:** 1 Waksman Institute of Microbiology, Rutgers, The State University of New Jersey, New Brunswick, New Jersey, United States; 2 Department of Genetics, Rutgers, The State University of New Jersey, New Brunswick, New Jersey, United States

## Abstract

The Anaphase Promoting Complex (APC) regulates the transition from metaphase to anaphase during cell division and is important to prevent defects in chromosome segregation. In a recent temperature sensitive genetic screen looking for further genes involved in fertilization, we isolated a new temperature sensitive allele of *mat-3 ( as49 )* . We also sequenced a previously identified *mat-3 ( or344 )* allele that did not previously have an annotated sequence. We determined that the *as49* allele was an Alanine to Threonine (A451T) mutation in the sixth exon and the *or344* mutation was a Leucine to Phenylalanine (L474F) mutation in the seventh exon. These locations of the mutant alleles are consistent with other previously annotated alleles that displayed the same metaphase to anaphase transition defect phenotype and further reinforce the importance of the tetratricopeptide repeats to mediate protein interactions.

**Figure 1. Characterization and analysis of mat-3 alleles:**
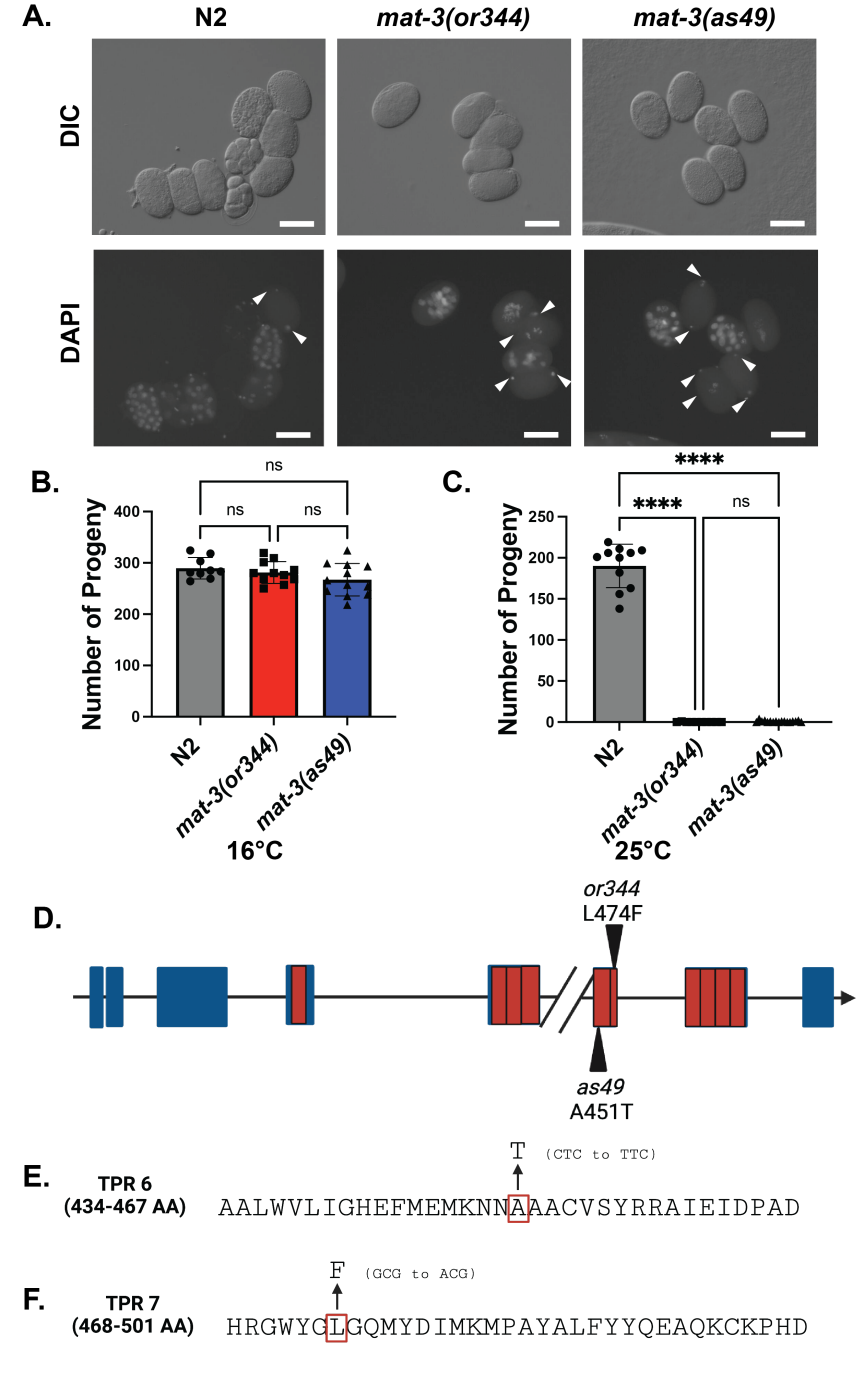
(A) DAPI and DIC images of wildtype, *mat-3 ( as49 ),* and *mat-3 ( or344 )* hermaphrodites *.* Arrowheads indicate the increased number of embryos where the sperm and oocyte DNA fail to proceed through meiosis. Scale bar equals 10mm. (B) Brood size of *mat-3 ( as49 ),* and *mat-3 ( or344 )* hermaphrodites at 16°C (n>=9, one-way ANOVA, ****p<0.0001) *.* (C) Brood size of *mat-3 ( as49 ),* and *mat-3 ( or344 )* hermaphrodites at 25°C (n>=10, one-way ANOVA, ****p<0.0001) *.* (D) Genomic locus of *mat-3* indicating the mutations that generated *mat-3 ( as49 )* and *mat-3 ( or344 )* . Red boxes indicate where the TPR domains are encoded. Each blue box indicates an exon. (E-F) Sequences of TPR domains 6 and 7. Red boxes indicate amino acids that were mutated and the change in DNA sequences are reflected in parentheses.

## Description

Uncovering new sterile mutations has been essential for understanding processes in fertilization, gamete activation, meiosis, and germline development. *C. elegans* are an ideal system for gene discovery due to their fully sequenced genome and plethora of genetic and molecular tools (Mei and Singson 2021). We have been conducting a forward genetic screen looking for sterile mutants with temperature sensitive phenotypes in *C. elegans.* Candidate mutations from this screen are fertile at the permissive temperature of 16°C but sterile at the restrictive temperature of 25°C. In this screen, as previously described, *sem-2 ( n1343 ) I; Is[Pelt-7::gfp; rol-6 ( su1006 )]* animals were mutagenized and their progeny were shifted to the restrictive temperature of 25°C, any sterile animals were then shifted back down to the permissive temperature of 16°C where they recovered fertility (Singaravelu et al. 2015) . This paper profiles the analysis of the sterile mutant that we determined was a new allele of the Anaphase Promoting Complex (APC) component *mat-3* . The new *mat-3* allele was named *as49* , additionally we sequenced a previously isolated temperature sensitive mutant of *mat-3 ( or344 )* to determine the mutation in the *or344* allele.

When shifted to the restrictive temperature as embryos or L1 larvae, the *as49* mutant isolated in this screen produced one cell embryos that failed to complete development and no live larval progeny. This was in contrast to wild-type embryos animals which quickly became multicellular and proceeded through development at the same conditions ( Figure 1A ). Brood size analysis of the *as49* mutant revealed that at the permissive temperature, there was no significant difference in number of progeny between the mutants and wild-type ( Figure 1B ). However, at the restrictive temperature, the wild-type control produced approximately 175 progeny and the *as49* mutant produced no live larval progeny ( Figure 1C ).

We next wanted to determine what chromosome the *as49* mutation was located on. Using previously described linkage mapping methods (Geldziler et al. 2005) , we determined that the *as49* mutation was on chromosome III. Analysis of F2 animals that were homozygous for the Dpy phenotype were shifted to the restrictive temperature for analysis of sterility. All 48 F2 animals which displayed the *dpy-18* phenotype were fertile. Therefore, we determined the *as49* was on chromosome III.

We observed that the *as49* mutants embryos frequently did not proceed past the one cell stage ( Figure 1A ) in contrast with the wild-type controls which displayed the expected progression of embryonic development in the uterus of *C. elegans* . This phenotype was reminiscent of mutants in the APC. Two previously identified APC genes were also located on chromosome III, *mat-3* and *emb-30 (Golden et al. 2000; Davis et al. 2002; Rappleye et al. 2002)* . We completed complementation testing and observed that the *as49* mutation and the *mat-3 ( or344 )* mutation failed to complement each other at restrictive temperature (100% sterile, n=48), indicating that they were likely the same gene. The *emb-30 ( tn377 )* mutants partially complemented *as49* phenotype at restrictive temperature (36% sterile, n=45). We hypothesize that as *emb-30* and *mat-3* both function in the APC complex to promote the ubiquitin mediated decay (Golden et al. 2000) , *as49 / emb-30 ( tn377 )* transheterozygotes may both have partially reduced function in the same pathway, this could be sufficient for the 36% sterility that was exhibited. Alternatively, the sterile progeny observed in the *as49 / emb-30 ( tn477 )* complementation testing could be due to some homozygous *emb-30 ( tn477 )* progeny still being produced. Cross progeny resulting from the successful cross of *as49 ; him-5 ( e1490 )* and *emb-30 ( tn377 )* was observed which decreases the likelihood of this occurring. Nonetheless, the incomplete penetrance of this phenotype compared to the 100% sterility of *as49 / mat-3 ( or344 )* likely indicates that *as49* was an allele of *mat-3* .

Now that we had identified the *as49* mutant was an allele of *mat-3 ,* we wanted to understand what the molecular change was that caused this phenotype and determine if this was the same as the previously isolated *or344* mutation which is also temperature sensitive. The *or344* mutation did not have a previously reported molecular characterization of the exact mutation. Using Sanger sequencing, we analyzed the *mat-3* locus from worms carrying the *as49* and *or344* alleles. The *as49* allele was an Alanine to Threonine (A451T) mutation in the sixth exon ( Figure 1D -E). The *or344* mutation was a Leucine to Phenylalanine (L474F) mutation in the seventh exon ( Figure 1D and F). Both of these mutations were in the tetratricopeptide repeats (TPR) which mediate protein-protein interactions during the metaphase to anaphase transition (Davis et al. 2002) . The location of these mutations is similar to many other *mat-3* alleles such as *ax70 , av26 ,* and *bs29 (Stein et al. 2007; Davis et al. 2008; Miller et al. 2016)* . The sequencing results of these two alleles further reinforce the importance of the TPR motifs in the metaphase to anaphase transition. Furthermore, these mutations continue to impart information about what molecular signatures can make a mutant temperature sensitive. Additional alleles of *mat-3* and many other genes provide valuable tools for analysis such as suppressor screens and structure function analysis.

## Methods

*C. elegans* strains were cultured as described in Brenner, 1974. L1 animals were shifted from 16°C to 25°C for phenotypic analysis.

The temperature sensitive forward genetic screen used to isolate *as49* was completed as described in (Singaravelu et al. 2015) . A synchronized L4 population of *sem-2 ( n1343 ) I; Is[Pelt-7::gfp; rol-6 ( su1006 )]* animals were mutagenized using EMS. *sem-2 ( n1343 ) I; Is[Pelt-7::gfp; rol-6 ( su1006 )]* animals were used to efficiently screen many animals. The *sem-2 ( n1343 )* mutants fail to lay embryos on the plate and will produce a bag of worm phenotypes when fertile. To further aid identification of sterile animals, this strain also has an *Is[Pelt-7::gfp; rol-6 ( su1006 )]* reporter, *elt-7* is first expressed during the L1 stage of larval development and illuminates the uterus of *sem-2 ( n1343 )* animals, therefore any sterile candidates would have a dark uterus. Following mutagenesis, the progeny were shifted to the restrictive temperature of 25°C, any sterile animals were then shifted back down to the permissive temperature of 16°C where they recovered fertility (Singaravelu et al. 2015) . After confirming the sterile phenotype, *sem-2 ( n1343 ) I; Is[Pelt-7::gfp; rol-6 ( su1006 )]* was crossed out of the background.

Brood size was completed as described in (Geldziler et al. 2005; Singaravelu et al. 2015) . In brief, one L4 animal was placed on an individual plate to lay progeny. This animal was transferred for its reproductive lifetime and all resulting progeny were counted.

Complementation was completed with *mat-3 ( or344 )* and *emb-30 ( tm377 )* (Yook et. al, 2004) . *as49 ; him-5 ( e1490 )* males were crossed to either *mat-3 ( or344 )* or *emb-30 ( tm377 )* hermaphrodites at 16°C as L4s, when we saw embryos on the plate, we shifted these plates to 25°C and looked for the presence of both male and hermaphrodite F1 progeny on the plate to determine if the cross was successful. The resulting F1 hermaphrodite progeny were then assessed for sterility after several days.

Linkage mapping was completed as described previously (Geldziler et al. 2005) . *as49 ; him-5 ( e1490 )* males were crossed with *dpy-18 ( e364 ), dpy-11(e224), dpy-13(e184),* or *tmC6* hermaphrodites. The resulting F1 hermaphrodite cross progeny were allowed to self-fertilize. Dpy F2 progeny were then cloned out to individual plates and assessed for fertility.

All imaging was conducted on day one adult hermaphrodites. L4s were picked the day before to ensure the age of the animals. Embryos were dissected out of one day old adults and either DIC imaging or DAPI staining was conducted.

DAPI staining was completed by washing animals with M9, fixing in cold methanol for 30 seconds, and washing with PBS. Animals or embryos were then transferred to a 2% Agarose pad with Vectashield mounting medium with DAPI (Vector Laboratories). The images were then captured on a Zeiss Universal microscope at the 40x objective with a ProgRes camera (Jenoptik) using ProgRes CapturePro software.

To determine the mutations in *mat-3 ,* we amplified the *mat-3* genomic sequence from lysed N2 , *mat-3 ( or344 ),* and *mat-3 ( as49 )* animals. Worms were lysed using single worm lysis buffer (50 mM KCl,10 mM Tris pH 8.2, 2.5 mM MgCl2, 0.45% NP-40, 0.45% Tween 20, and 10mg/mL Proteinase K) on the following PCR program, 60°C for one hour, 95°C for 15 minutes, and then held at 4°C (Ahringer et al. 2006) . The DNA was PCR amplified in two overlapping ~4kb fragments. Primers were then designed for every 500 bp along the length of the gene to be used for Sanger sequencing. The results from sequencing were then assembled and compared to the WBcel235/ce11 genome assembly as well as N2 to confirm that this was not a background mutation.

## Reagents

Table 1. Strains used in this paper

**Table:** 

Strain	Genotype	Available From
N2	Wild-type	Caenorhabditis Genetics Center
HY601	*mat-3 ( or344 )* III	Caenorhabditis Genetics Center
AD322	*mat-3 ( as49 )* III	Singson Lab
AD351	*mat-3 ( as49 )* III; *him-5 ( e1490 ) V *	Singson Lab
AD98	*sem-2 ( n1343 ) I; Is[Pelt-7::gfp; rol-6 ( su1006 )] *	Singson Lab
DG627	*emb-30 ( tn377 ) III *	Caenorhabditis Genetics Center
CB61	*dpy-5(e61) I*	Caenorhabditis Genetics Center
FX19668	*tmC6 [dpy-2(tmIs1189)] II*	Caenorhabditis Genetics Center
CB364	*dpy-18 ( e364 ) III *	Caenorhabditis Genetics Center
CB224	*dpy-11(e224) V*	Caenorhabditis Genetics Center
CB184	*dpy-13(e184) IV*	Caenorhabditis Genetics Center

Table 2. Primers used in this paper

**Table:** 

ggtttctcacacacacaatc	KM47	mat-3 Forward Primer Fragment 1
GGCTAACGGATAACTACGTA	KM48	mat-3 Reverse Primer Fragment 1
TACGTAGTTATCCGTTAGCC	KM49	mat-3 Forward Primer Fragment 2
gtcgctacctacacaaactc	KM50	mat-3 Reverse Primer Fragment 2
ccccgtgtatatcgatttttc	KM51	mat-3 sequencing primer
CACAAAGTTTGGACTGCTAG	KM52	mat-3 sequencing primer
GAATGACGCTGAAGGAATCG	KM53	mat-3 sequencing primer
CCCCGTGACCTTTTTCAATT	KM54	mat-3 sequencing primer
GCATATTTCCACGTTCTCCC	KM55	mat-3 sequencing primer
CAGCCAAATTTCCACGTGGA	KM56	mat-3 sequencing primer
GGATGGGTGACTGGTTCATG	KM57	mat-3 sequencing primer
CGGCTAATTCGGCTAATTCG	KM58	mat-3 sequencing primer
GGCCGTTGTTAGCCGAATTC	KM59	mat-3 sequencing primer
CCGAACATTTTGGGATTTGG	KM60	mat-3 sequencing primer
CAAACTGTGTTCCTGTTGAT	KM61	mat-3 sequencing primer
CAGCCAACTACCACGCAATC	KM62	mat-3 sequencing primer
CGATCCAGCAGATCACCGTG	KM63	mat-3 sequencing primer
CGGTAAAATTTGAGGGTTCA	KM64	mat-3 sequencing primer
